# Effect of parathyroidectomy on stone recurrence in primary hyperparathyroidism: A systematic review

**DOI:** 10.1007/s00508-026-02733-9

**Published:** 2026-03-24

**Authors:** Victoria Jahrreiss, Ozan Yurdakul, Julian Veser, Christian Seitz

**Affiliations:** Department of Urology, Medical University of Vienna, Vienna General Hospital, Vienna, Austria

**Keywords:** Primary hyperparathyroidism, Parathyroidectomy, Urolithiasis, Recurrence hypercalciuria

## Abstract

**Background:**

Primary hyperparathyroidism (pHPT) is infrequently associated with calcium-containing kidney stones, despite hypercalciuria. Parathyroidectomy (PTX) is the only curative treatment of the metabolic disorder and is indirectly regarded as prophylaxis of recurrence of stone formation. The aim of the systematic review was to evaluate the impact of PTX on stone recurrence and to identify possible predictors of recurrent stone formation.

**Methods:**

Following PRISMA guidelines, a systematic PubMed search was conducted through April 2025. Eligible studies including adults with successfully surgically treated pHPT, documented nephrolithiasis and a follow-up of at least 12 months were analyzed.

**Results:**

A total of 13 studies (2 prospective cohorts, 10 retrospective cohorts, 1 randomized controlled trial, RCT) comprising more than 8000 patients met the inclusion criteria. After PTX, recurrence rates in prospective studies ranged from 0–30% among stone formers, while retrospective series showed a wider range (between 0% and 58%). Registry data indicated that in patients with a history of nephrolithiasis, the recurrence risk seems higher shortly after PTX compared with conservative management of pHPT but decreases substantially with each subsequent year. In patients without a stone history, de novo stone formation was rare; in the RCT, recurrence occurred in 0% after PTX versus 4% under observation. Consistent predictors of recurrence included persistent hypercalciuria, multiple preoperative stone episodes, hypocitraturia, elevated body mass index, and male sex.

**Conclusion:**

The use of PTX significantly reduces the long-term risk of stone recurrence but does not eliminate it entirely. In patients with a history of nephrolithiasis, short-term risk may be elevated after PTX but declines markedly over time.

Individualized follow-up including regular assessment of serum calcium, parathyroid hormone, renal function, and 24‑h urinary parameters, together with preventive measures where indicated, such as adequate fluid intake, dietary counselling, potassium citrate or thiazide therapy, is recommended, particularly in patients with persistent risk factors.

Decisions regarding surgical treatment of existing kidney stones should be individualized based on stone size, location, symptoms, infection risk as well as the overall patient risk profile and life expectancy.

## Introduction

Nephrolithiasis is among the most frequent and clinically relevant renal manifestations of primary hyperparathyroidism (pHPT). Reported prevalence rates range from about 10% [1] to 20% [2] depending on the study population and diagnostic methods. Kidney stones are significantly more common in patients with pHPT compared with the general population, with some studies showing a fourfold increased risk [3].

Many stones are asymptomatic and are discovered through routine imaging [4].

Stone formation is driven by multiple mechanisms: Besides signalling bones to release calcium, parathyroid hormone (PTH) promotes (together with vitamin D) intestinal calcium absorption and causes calcium reabsorption in the kidney tubules, increasing serum calcium. This leads to a higher filtered load of calcium at the kidney glomerulus and therefore increased phosphate and calcium excretion in the urine resulting mainly in hypercalciuria. Hypercalciuria promotes urinary supersaturation with calcium oxalate and calcium phosphate, facilitating crystal formation. Additional metabolic abnormalities (hypocitraturia, hyperoxaluria, hyperuricosuria) as well as structural damage in the kidneys from previous stones can further predispose to recurrent stone formation [5, 6].

Beyond nephrolithiasis, pHPT can be manifested as other renal complications, including nephrocalcinosis and impaired kidney function. Nephrocalcinosis, characterized by calcium deposition in the renal parenchyma, has been reported in up to 5–10% of pHPT patients and may contribute to progressive renal dysfunction. Chronic hypercalcemia and hypercalciuria can directly impair renal tubular function, leading to a decreased glomerular filtration rate in a severe or longstanding disease. While this review focuses specifically on kidney stone recurrence and outcome, a variety of renal manifestations influence the clinical decision-making regarding parathyroidectomy and highlight the systemic nature of untreated pHPT.

Parathyroidectomy (PTX) is the only definitive treatment of pHPT [7]. Restoring normocalemia, PTX is expected to reduce urinary calcium excretion and thereby to diminish the risk of recurrent nephrolithiasis; however, recurrence can still occur in a subset of patients after successful surgery. In some cases, stone disease precedes the diagnosis of pHPT by several years, suggesting that idiopathic hypercalciuria or other persistent risk factors may play a role [8, 9].

The aim of this systematic review is to synthesize the current evidence on stone recurrence after PTX in patients with pHPT and a history of nephrolithiasis, to compare outcomes with nonsurgical management and to identify predictors of recurrence.

## Methods

### Search strategy

This study followed the Preferred Reporting Items for Systematic Reviews and Meta-Analyses (PRISMA) guidelines [10].

A systematic search of PubMed was conducted up to 1 April 2025. Search terms included: (“primary hyperparathyroidism” OR “pHPT”) AND (“parathyroidectomy” OR “PTX”) AND (“urolithiasis” OR “nephrolithiasis” OR “kidney stones”) AND (“recurrence” OR “stone recurrence” OR “outcome”). Only human studies in English or German were included.

### Inclusion and exclusion criteria

#### Inclusion criteria

1. Included adults with biochemically confirmed pHPT and documented nephrolithiasis before intervention.
2. Reported postoperative stone recurrence rates after PTX.
3. Had ≥ 12 months of follow-up.
4. Included ≥ 10 patients.

#### Exclusion criteria

Case reports, reviews, studies without recurrence data, or without separate data for pHPT stone formers.

### Data extraction and quality assessment

The two authors VJ and OY independently extracted study design, sample size, follow-up duration, recurrence rates, recurrence definitions, and predictors.

### Outcomes

Primary outcome: postoperative stone recurrence rate.

Secondary outcomes: comparison with non-PTX management, time to recurrence, recurrence-free survival, and predictors.

## Results

The search identified 512 records, of which 421 remained after removal of duplicates. After screening titles and abstracts, 45 full-text articles were assessed for eligibility and 13 studies met all inclusion criteria. These comprised 2 prospective studies [11, 12], 10 retrospective studies [9, 15–22, 26] and 1 randomized controlled trial (RCT) [13]; with a total of > 8000 patients with pHPT and documented nephrolithiasis (Table 1). The median follow-up duration ranged from 24–84 months. Definitions of recurrence varied, with most studies including symptomatic stone events or radiological detection of new calculi; a minority required urological intervention as part of the definition.

**Table 1:** Characteristics of studies included in the systematic review evaluating kidney stone recurrence after parathyroidectomy in primary hyperparathyroidism

Study	Design	Stone patients	Follow-up (years)	Stone recurrence
Mollerup 2002 [9]	Retrospective	167 SF PTX	5	58%
		506 NSF PTX		3%
Mollerup 1999 [12]	Prospective	107 SF PTX	5	30%
Rowlands 2013 [14]	Retrospective	65 SF PTX	4.3	1.5%
		65 NSF PTX	5.1	0%
		65 ISF NPTX	4.3	25%
Lui 2022 [26]	Retrospective	76	3	7%
Seib (2021)	Retrospective	486 SF PTX	5	17.9%
		2447 NSF PTX		2.9%
		489 SF NPTX		16.4%
		4201 NSF NPTX		2.6%
Seib 2022 [22]	Retrospective	1154 SF PTX	5.6	30.5%
		4473 NSF PTX	5	4.7%
		2886 SF NPTX		18%
		35261 NSF NPTX		2.6%
Charles 2021 [17]	Retrospective	30 SF PTX	4 (2–6)	5–15%
Islam 2020 [16]	Retrospective	69 SF PTX	4 ± 2.9	23%
Sorensen 2012 [15]	Retrospective	40 SF PTX	2	23%
		50 NSF PTX		0%
Silverberg 1999 [11]	Prospective	12 SF PTX	Up to 10 years	0%
		8 SF NPTX		75%
Rao 2004 [13]	RCT	23 NSF PTX	3.5 (2–4.5)	0%
		25 NSF NPTX		4%
Huang 2020 [20]	Retrospective	334 SF PTX	15	37%
		918 SF observation		29%
Axelsson 2020 [19]	Retrospective	547 SF PTX	1.2	HR, 0.61; 95% CI, 0.48–0.78
		437 SF NPTX	4.6	0.89 (95% CI, 0.76–1.06)

*ISF* idiopathic stone formers, *NPTX* no parathyroidectomy, *NSF* no stone formers, *PTX* parathyroidectomy, *SF* stone formers, *HR* hazard ratio, *CI* confidence interval, *RCT* randomized controlled trial

Prospective studies [11, 14] demonstrated low recurrence rates after PTX. Silverberg et al. [11] followed 20 symptomatic patients with kidney stones for up to 10 years; none of the 12 patients who underwent PTX developed recurrent stones, compared with the 6 (75%) of the 8 patients who did not undergo surgery. Among asymptomatic pHPT patients in the same study, no recurrences occurred regardless of treatment. Rowlands et al. [14] found a recurrence rate of 1.5% in 65 surgically treated stone formers over a median follow-up of 4.3 years, compared with 25% in idiopathic stone formers. Notably, no new stones developed in pHPT patients without a history of stones.

In the randomized controlled trial, Rao et al. [13] included 81 asymptomatic pHPT patients (53 with PTX, 28 under observation) within the observation period of 3.5 years, no recurrences occurred after surgery, compared to 4% in the observation group.

Retrospective series [9, 15, 22, 26] reported more variable recurrence rates. Sorensen et al. [15] examined 40 stone and 50 non-stone formers undergoing PTX, with a median follow-up of 24 months. Recurrence occurred in 23% with stones but in none without. Male sex (adjusted odds ratio, aOR, 20) and higher body mass index (BMI, aOR 1.23) were independent predictors of recurrence. No metabolic biochemical parameter predicted recurrence. Similarly, Islam et al. [16] reported a recurrence rate of 23% among 69 stone formers during a mean follow-up of 4 ± 2.9 years; younger age at surgery (51 vs. 60 years) was a significant predictor, and persistent hypercalciuria was observed in 54% of patients postoperatively. Charles et al. [17] found recurrence rates of 5–15% among 30 pHPT stone formers, along with significant reductions in urinary calcium saturation indexes postoperatively; Vestergaard et al. [18] conducted a cohort study reporting that patients with pHPT who had a history of kidney stones (previous stone formers) were more than twice as likely to undergo surgery than conservative treatment. After diagnosis, the risk of developing kidney or urinary tract stones remained higher in the surgery group. The hazard ratio (HR) for stone events after diagnosis in the surgery group compared to the conservative group was 1.87 (95% CI: 1.30–2.68) and 9% of surgically treated patients had kidney stones after diagnosis versus 3% of non-surgically treated patients. More episodes of kidney stones occurred after diagnosis among those who had surgery than those who did not, and a history of stones was a strong predictor for recurrence (HR for recurrence if prior event: 8.62, 95% CI: 6.51–11.43).

Axelsson et al. [19] investigated the outcomes before and after surgery of 6934 patients. During follow-up, they found the risk of kidney stones almost 4 times higher in patients with pHPT than in controls (unadjusted HR 3.65; 95% CI 3.27–4.08). Among patients who underwent PTX, the risk of kidney stone after surgery was significantly lower compared to before surgery (HR 0.61; 95% CI 0.48–0.78). In their study data on pHPT-related symptoms, serum calcium, or PTH were not available, preventing the assessment of pHPT severity. Patients who underwent PTX had a significantly lower risk of recurrent kidney stones than those who were managed conservatively. This reduction is substantial, with the risk decreasing by about 23% (HR 0.77) compared to conservative treatment and by 39% (HR 0.61) when comparing after PTX to before PTX within the same patients. In the Axelsson cohort, the hazard ratio for kidney stone events comparing conservative management with parathyroidectomy was 0.89 (95% CI 0.76–1.06), indicating no statistically significant reduction in risk between treatment strategies during the observed follow-up period [19].

Mollerup et al. [12] observed a 30% recurrence rate within 5 years in 107 stone formers after successful PTX and normocalcemia with a recurrent rate comparable to idiopathic stone formers. In a large retrospective cohort, Mollerup et al. [9] studied 674 surgically verified pHPT patients compared to matched controls and found a 58% reduction in recurrence risk after PTX. Patients with prior stones had a 27-fold higher risk of postoperative recurrence compared with controls, and more than 10 years after surgery, recurrence risk approached that of the control population, as shown by Mollerup et al. [9] and Vestergaard et al. [18]. Data from registries and RCTs reinforced the benefit of PTX. Huang et al. [20] reported recurrence-free survival after PTX of 82.4%, 70.9%, and 62.8% at 5, 10, and 15 years, respectively, compared to 74.4%, 56.3%, and 49.5% before surgery (*p* < 0.0001). Seib et al. published two series [21, 22]. The first [21] investigated the association of PTX with 5‑year clinically significant stone events in patients with pHPT. The unadjusted incidence of kidney stone events within 5 years was 2.9% in patients without a history of kidney stones who underwent PTX within 1 year of diagnosis and 2.6% in those observed. After inverse probability weighted adjustment using propensity scores, there was no difference in the adjusted odds of a kidney stone event in years 2–5 after PHPT diagnosis among patients who were treated with PTX versus nonsurgery (OR 1.16; 95% CI 0.84–1.60). In the second registry study [22] analyzing patients with a prior history of nephrolithiasis, the unadjusted incidence of at least one recurrent stone event was higher after surgery compared to conservative management (30.5% vs. 18.0%; mean follow-up 5.6 vs. 5.0 years). After adjustment, PTX was associated with an increased risk of stone recurrence (HR 1.98, 95% CI 1.56–2.51); however, the risk declined by approximately 20% with each additional year of follow-up (HR per year 0.80, 95% CI 0.73–0.87). Among patients without a history of nephrolithiasis, the unadjusted incidence of stone events was low in both groups (4.7% after PTX vs. 2.6% with without surgery; mean follow-up 8.1 vs. 6.2 years). In this subgroup, adjusted analyses revealed no significant difference in risk (HR 1.14, 95% CI 0.76–1.71) [22]. The rate of stone events per 1000 patients in the PTX group declined from 224 in the year before surgery to 144 in the year after and to 28 at 5 years; the observation group showed smaller declines (133 to 60 to 41 per 1000 patients, respectively). Predictors of recurrence included history of stone formation, high BMI (> 30), and male sex [15]. Across several studies, patients without a stone history before surgery did not develop new stones after PTX, even with follow-up durations up to 10 years [14, 15].

## Discussion

This systematic review demonstrates that successful PTX substantially reduces the risk of recurrent kidney stones with relative risk reduction up to 85% compared to patients without surgery.

Prospective cohorts and RCTs reported recurrence rates between 0–30% [11–13], whereas retrospective studies showed wider data between 0 and 58% [9, 11], likely reflecting differences in recurrence definitions, patient selection, techniques used for detecting possible kidney stones and in follow-up duration. Importantly, no new stones were revealed after PTX in patients without prior nephrolithiasis. The degree of hypercalciuria alone does not determine stone formation in pHPT patients as it is observed in non-stone formers underlining that stone formation is a multifactorial systemic disorder. Therefore, postoperatively stone forming risk may remain increased from other contributing factors. The current evidence on the benefit of PTX on stone recurrence is still controversial. Reasons are the lack of more than one RCT, and the findings that the risk of stone recurrence is decreasing with time. Different results may be influenced by the length of follow-up period. Additionally, the determination of primary outcomes may differ substantially as new stone formation can be determined by radiological interventions in asymptomatic patients or symptomatic recurrence or presentation to an emergency department, potentially underestimating the recurrence rates. The low rate of recurrence rates in the study from Rowlands et al. [14] may be further explained by using symptomatic recurrence rates in patients returning to hospital for further radiological work-up in contrast to studies with routine follow-up radiological imaging for stone detection. Underestimation of stone recurrence may be attributable to the use of ultrasound and conventional radiography instead of computed tomography [23, 24].

### Practical advice

An important consideration could be the misinterpretation of preoperatively asymptomatic (undiagnosed, persistent) kidney stones as “recurrent” stones becoming “symptomatic” following PTX. Selberherr et al. [24] demonstrated that multidetector computed tomography (MDCT) detects stones in 53.5% of patients with asymptomatic pHPT, compared to only 14.1% detected by ultrasound. These silent stones, if not identified preoperatively through systematic imaging, may later be incorrectly classified as new recurrences, thereby inflating recurrence rates and underestimating the true benefit of PTX. Zabolotniuk et al. [25] further emphasized that preoperative imaging protocols can identify previously undiagnosed stones, improving the accuracy of recurrence assessments. Preoperative imaging, preferably with non-contrast computed tomography should be considered for patients with pHPT before undergoing PTX. This approach not only provides accurate baseline assessment but also informs decisions regarding concurrent stone treatment. Persisting renal calculi after successful PTX should be reassessed once normocalcemia has been restored, typically after 3–6 months. Stones that remain stable, asymptomatic, and small may be managed conservatively, whereas enlarging stones, symptomatic stones, or those with infection risk should undergo standard urological treatment according to contemporary stone guidelines. A careful urological monitoring with ongoing controls of the kidney function is strongly recommended in patients with persistent kidney stones.

Reports, from Mollerup et al. [9], demonstrating substantial risk reduction after PTX. Long-term registry data from Seib et al. [22] and Huang et al. [20] show that stone recurrence-free survival improves progressively after surgery, and the absolute benefit increases over time compared to observation. These data support the causal role of pHPT in stone formation.

The persistence of recurrences in some patients, as observed by Islam et al. [16] and Sorensen et al. [15], underscores the contribution of non-PTH-related risk factors. Although PTX removes some metabolic drivers of stone formation, residual recurrence risk may be due to persistent idiopathic hypercalciuria, which can precede the diagnosis of pHPT. Structural sequelae of prior stones, such as papillary damage or urinary tract strictures, may predispose to recurrence regardless of metabolic correction. Persistent hypocitraturia may also maintain lithogenic potential despite normocalcemia. Additionally, recurrent (or very rarely recurrent pHPT) can reintroduce hypercalciuria.

Hydration, dietary modification, and thiazide teatment are considered less effective than PTX in recurrence prevention [17]; however, persistent hypercalciuria and hypocitraturia are consistent risk factors, warranting postoperative 24‑h urine evaluations. Multiple preoperative stone events also increase the recurrence risk [9]. Male sex and high BMI are nonmetabolic predictors in some cohorts [26, 27] and high-risk patients may benefit from targeted interventions such as potassium citrate, thiazides, and dietary counselling, with periodic imaging Postoperative follow-up imaging may be performed after 6–12 months and subsequently at individualized intervals (e.g., annually), depending on residual stone burden and metabolic risk profile for early detection of asymptomatic stones.

Seib et al. [22] reported greater and more sustained reductions in stone events after PTX compared to observation. Overall, these findings of Seib et al. [22] indicate that while patients with previous nephrolithiasis initially experience a higher recurrence risk following PTX compared to conservative management, this risk diminishes substantially over time. In patients without a stone history, no significant difference between treatment strategies was observed.

Despite evidence that pHPT is an important and treatable cause of kidney stones, screening rates remain low, representing a significant gap in urological and endocrine practice. Lui et al. [28] demonstrated that only 4.9% of patients with nephrolithiasis undergo screening for hyperparathyroidism (at least measuring serum calcium once at the time of treating nephrolithiasis!), yet 13.5% of those screened are diagnosed with pHPT. The consequences of missed diagnosis extend beyond recurrent stone disease to include progressive bone loss, potential cardiovascular complications, and impaired quality of life from untreated hypercalcemia [28]. Ganesan et al. [29] showed that systematic screening protocols can substantially improve pHPT detection rates among stone formers, leading to timely intervention that reduces both stone recurrence and other complications of pHPT. This underlines the importance of metabolic testing, at least in high risk and recurrent stone formers.

### Limitations

The heterogeneity of definitions of recurrence and detection methods, predominance of retrospective designs, and limited long-term data restrict the strength of conclusions. Data on pHPT-related symptoms, serum calcium, or parathyroid hormone were not available in all studies, preventing the assessment of pHPT severity. Few studies examined the effect of postoperative metabolic interventions, and most lacked standardized follow-up imaging.

## Future research directions

Future studies should standardize recurrence definitions, evaluate postoperative preventive measures, and assess cost-effectiveness of intensive follow-up in high-risk subgroups.

## Conclusion

Successful parathyroid surgery restoring normocalemia significantly reduces the long-term risk of stone recurrence but does not eliminate it entirely. In the short term, the risk of recurrent kidney stone events after PTX is higher than with conservative management of PHPT. Over the long term, however, this risk decreases significantly following PTX. Patients without a history of kidney stones, do not develop kidney stones during follow-up. Patients with persistent metabolic abnormalities such as hypercalciuria, hypocitraturia or rarely, persistent or recurrent hyperparathyroidism as well as those with multiple prior stones or adverse demographic risk factors require closer follow-up. Long-term monitoring should include periodic assessment of serum calcium, parathyroid hormone levels, renal function, and, when indicated, 24‑h urine metabolic evaluation. Targeted preventive measures such as adequate hydration, dietary counselling, citrate supplementation, or thiazide therapy may be required to sustain the benefit of surgery.

**Fig. 1:**
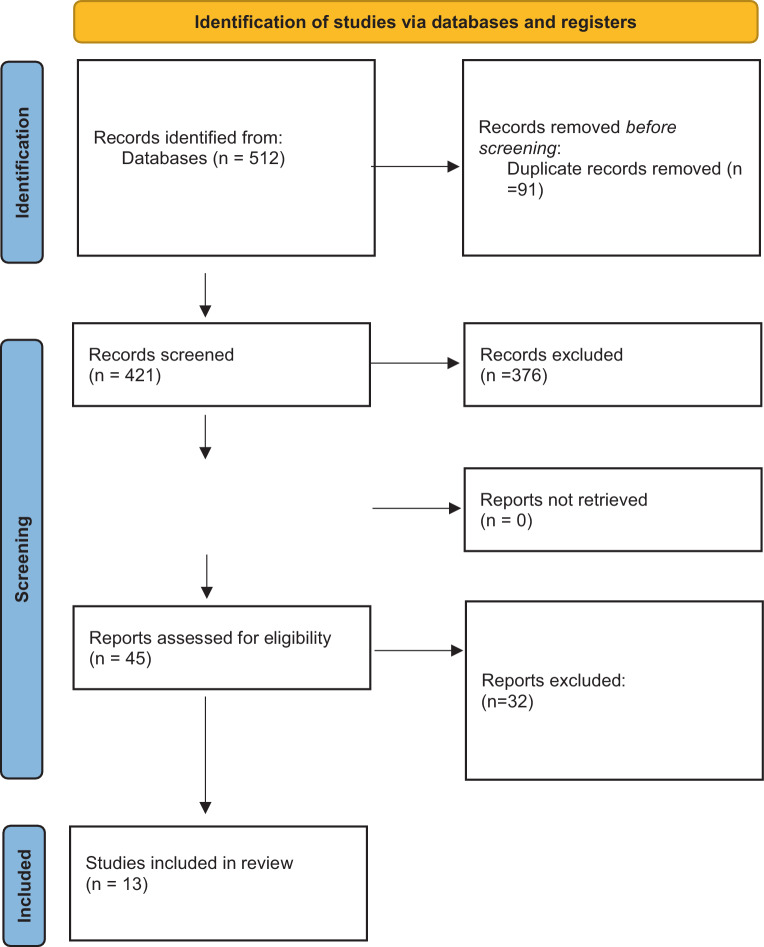
PRISMA flow diagram showing study selection process. Duplicate removal and screening followed PRISMA 2020 recommendations
